# Surgical outcomes analysis in patients with uncomplicated acute type A aortic dissection: a 13-year institutional experience

**DOI:** 10.1038/s41598-020-71961-4

**Published:** 2020-09-10

**Authors:** Chun-Yu Lin, Lai-Chu See, Chi-Nan Tseng, Meng-Yu Wu, Yi Han, Cheng-Hui Lu, Feng-Chun Tsai

**Affiliations:** 1 Department of Medicine, College of Medicine, Chang-Gung University, Taoyuan, Taiwan; 2 Department of Cardiothoracic and Vascular Surgery, Chang-Gung Memorial Hospital, Linkou Medical Centre, 5 Fu-Shing Street, Kwei-Shan, Taoyuan, 333 Taiwan; 3 Department of Cardiothoracic and Vascular Surgery, Chang-Gung Memorial Hospital, Tucheng Branch, New Taipei, Taiwan; 4 Department of Public Health, College of Medicine, Chang-Gung University, Taoyuan, Taiwan; 5 Biostatistics Core Laboratory, Molecular Medicine Research Centre, Chang-Gung University, Taoyuan, Taiwan; 6 Division of Rheumatology, Allergy and Immunology, Linkou Medical Centre, Chang-Gung Memorial Hospital, Taoyuan, Taiwan; 7 Department of Cardiology, Chang-Gung Memorial Hospital, Linkou Medical Centre, Taoyuan, Taiwan

**Keywords:** Cardiology, Diseases

## Abstract

This retrospective study aimed to clarify the short-term and mid-term outcomes of and prognostic factors for patients who underwent surgical repair for uncomplicated acute type A aortic dissection (ATAAD). Between January 2007 and June 2019, 603 consecutive patients underwent ATAAD repair at our institution. According to patients’ preoperative presentations and imaging studies, uncomplicated ATAAD was found in 276 (45.8%) patients by excluding preoperative complicated factors. Patients with uncomplicated ATAAD were classified into the survivor (n = 243) and non-survivor (n = 33) groups. Clinical features, surgical information, and postoperative complications were compared. Three-year survival and freedom from reoperation rates for survivors were analyzed using the Kaplan–Meier actuarial method. The in-hospital surgical mortality rate of uncomplicated ATAAD patients was 11.9%. The non-survivor group had a higher rate of postoperative malperfusion-related complications, and a multivariate analysis revealed that repeat surgery, retrograde cerebral perfusion, and intraoperative extracorporeal membrane oxygenation support were predictors of in-hospital mortality. In the survivor group, 3-year cumulative survival and freedom from aortic reoperation rates were 89.6% (95% confidence interval [CI] 84.8–92.9%) and 83.1% (95% CI 76.8–87.7%), respectively. In conclusion, uncomplicated and complicated ATAAD rates were similar; the short-term and mid-term surgical outcomes in patients with uncomplicated ATAAD were generally acceptable.

## Introduction

Acute type A aortic dissection (ATAAD) is a cardiovascular emergency associated with high morbidity and mortality rates. It is challenging for cardiothoracic surgeons because of the complex anatomy of the aorta and prevalence of preoperative complications. In previous studies, complicated preoperative conditions, such as the presence of hemodynamic instability, requirement for cardiopulmonary resuscitation (CPR), end-organ malperfusion, and haemopericardium with cardiac tamponade, were recognized as adverse factors for surgical outcomes of ATAAD patients^1–4^. Among these high-risk patients, the in-hospital mortality rates were reported to be 36–54%, which was approximately twice that of the majority of ATAAD population^5,6^. Those with uncomplicated ATAAD comprise a relatively stable subgroup; however, their characteristics and surgical results seem underreported. Furthermore, a consensus regarding the definition of uncomplicated ATAAD is lacking. In a previous study reported by Czerny et al., the uncomplicated ATAAD was defined as the absence of preoperative malperfusion regardless of other established risk factors for in-hospital mortality^3^. In another study reported by Piccardo et al., which was conduced with a more extended definition, patients without preoperative CPR, neurological deficit, and mesenteric ischemia were defined as uncomplicated^7^. However, it only investigated the octogenarian population with a small sample size. In the present study, by excluding most of the preoperative complicated factors to obtain a study group of ATAAD patients with the lowest surgical risk, we generated a retrospective analysis of the experiences of an individual aortic surgery centre and aimed to clarify the short-term and mid-term outcomes of and prognostic factors for patients who underwent surgical repair for uncomplicated ATAAD.

## Methods

### Definitions

After referencing the definitions in previous literature^1–4^, complicated ATAAD was defined as presenting with preoperative shock status, organ malperfusion, haemopericardium, ventilator support, requirement for CPR, or a combination of these conditions before undergoing ATAAD repair surgery. Shock was defined as systolic blood pressure (SBP) < 90 mmHg or requirement for inotropic medication. Organ malperfusion syndrome was defined according to evidence of a lack of blood flow to the defined organ system on imaging studies in accordance with clinical symptoms regarding the involved organ. Organ malperfusion syndromes included cerebral, spinal, myocardial, limb, renal, and mesenteric malperfusions^8^. Without symptoms of malperfusion, the independent radiographic evidence of dissection flap in the arterial branch vessels was not considered as malperfusion syndrome. In contrast, patients who did not present any features associated with complicated ATAAD were classified as having uncomplicated ATAAD.

### Patient enrollment and preoperative management

The study protocol was approved by the Chang-Gung Medical Foundation Institutional Review Board (No.202000118B0), and all methods were performed in accordance with the relevant guidelines and regulations. With approval from the Chang-Gung Medical Foundation Institutional Review Board, the need for informed consent was waived due to the retrospective nature of the study. Overall, 603 consecutive adult patients underwent emergency ATAAD repair at our institution between January 2007 and June 2019. All patients were diagnosed via helical computed tomography to confirm ATAAD in the emergency department, and the extent of aortic dissection, presence of organ malperfusion, and haemopericardium were analyzed. When the diagnosis of ATAAD was confirmed, patients were emergently transferred to the operating room within 30 min without delay, irrespective of whether the preoperative condition was complicated or uncomplicated. If patients presented with hypertension or tachycardia before surgery, then their hemodynamics were stabilised with intravenous beta-blockers to maintain SBP < 120 mmHg and a heart rate of 60–70 bpm, according to the 2010 American College of Cardiology/American Heart Association guidelines for thoracic aortic disease^9^. When patients presented with shock, medical resuscitation was applied first, including intravenous fluid supplementation and inotropic infusion. Rescue procedures, including pericardiocentesis and subxiphoid pericardiotomy, were performed if unstable hemodynamics persisted and cardiac tamponade was found by on-site echocardiography. For refractory hemodynamic instability or cardiac arrest, CPR was performed. All management strategies were performed on an emergency basis.

**Figure 1:**
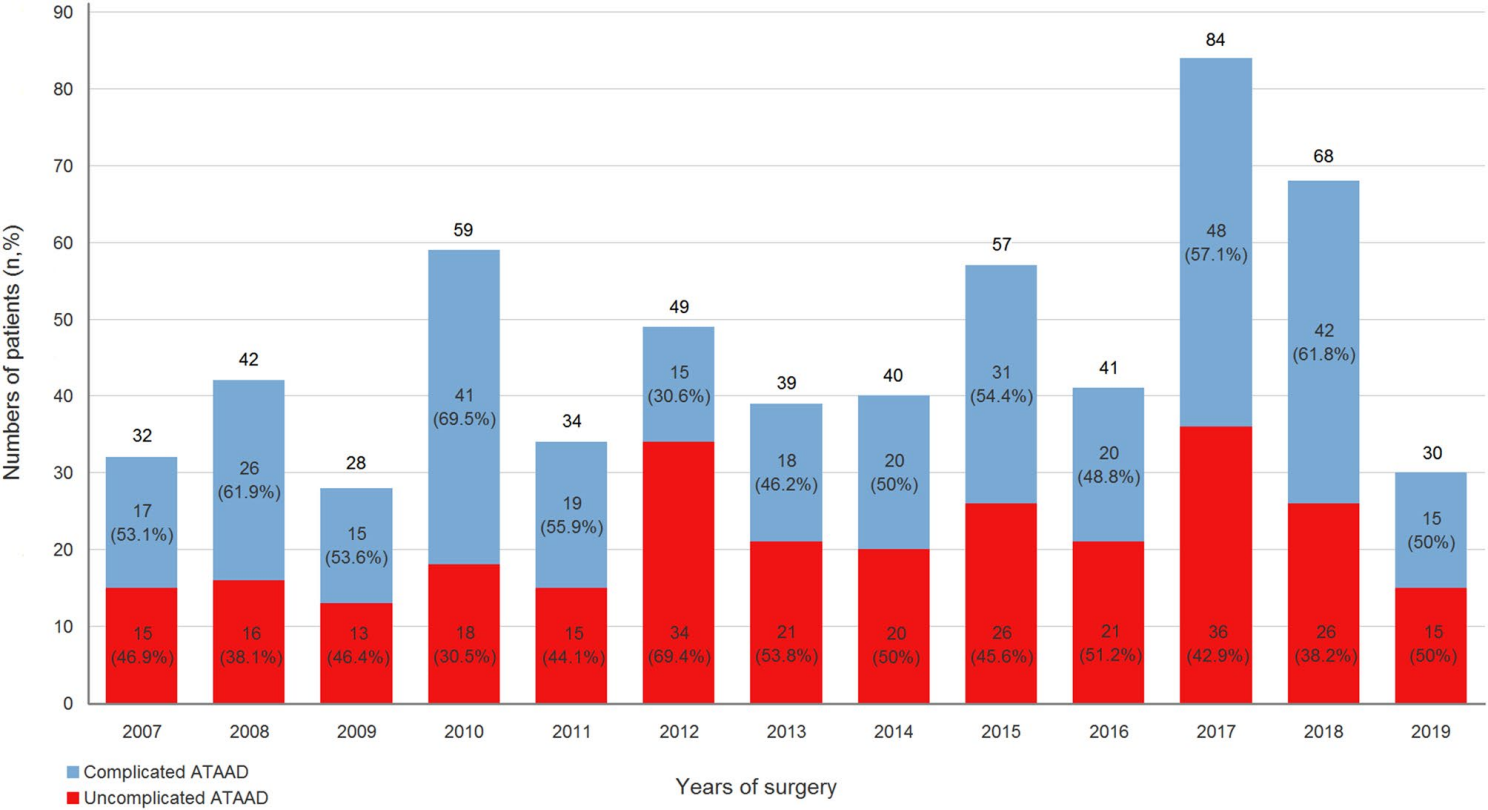
Distribution of ATAAD patients from January 2007 to June 2019. ATAAD, acute type A aortic dissection.

**Figure 2:**
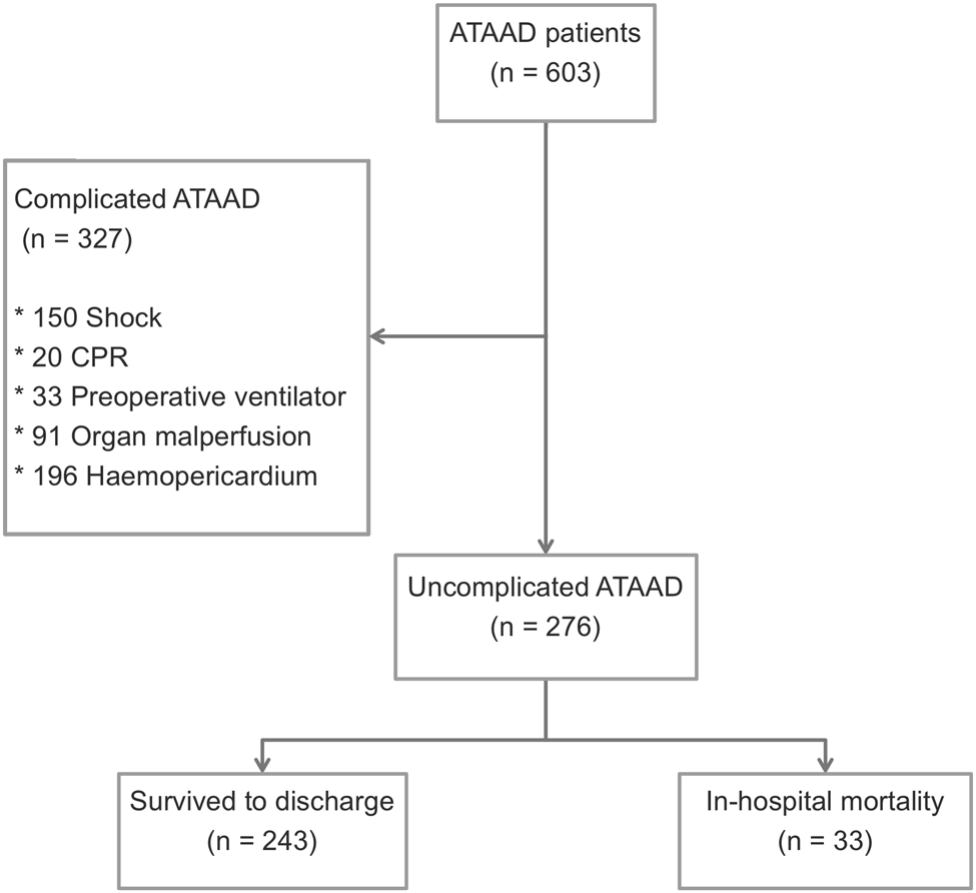
Enrollment and allocation of ATAAD patients during the study period. ATAAD, acute type A aortic dissection.

The overall number of patients per year who underwent ATAAD repair surgery and the number of that with uncomplicated type per year during the study period are illustrated in Fig. 1. As illustrated in Fig. 2, 327 patients with complicated ATAAD were excluded, including 150 with shock, 20 with CPR, 33 with preoperative ventilator support, 91 with organ malperfusion, and 196 with haemopericardium. Given that a single patient can have more than one etiologies of complicated ATAAD, the sum of all these categories was not equal to 327. A total of 276 patients who underwent aortic repair surgery for uncomplicated ATAAD were included. The included patients were dichotomised into the following groups according to whether they survived to discharge: survivor (n = 243) group and non-survivor (n = 33) group.

### ATAAD repair procedures and postoperative care

The general principles of aortic repair procedures are detailed in previous studies reported by this institute^10,11^. For uncomplicated ATAAD patients who were considered relatively stable, double arterial cannulation with antegrade cerebral perfusion (ACP) strategy was usually implemented. The right axillary and femoral arteries were cannulated with an 8-mm ring-reinforced polytetrafluoroethylene graft and connected with a Y-shape circuit. Following sternotomy, the right atrium was cannulated and cardiopulmonary bypass (CPB) with deep hypothermia was initiated. In general, the dissected aorta was replaced with a Dacron prosthetic graft based on the location of the entry tear and preoperative presentation. The proximal anastomosis was usually performed first, followed by open distal anastomosis under circulatory arrest. During circulatory arrest, the femoral arterial flow was temporarily suspended and selective ACP through the right axillary artery was used. Concomitant aortic root replacement with a composite Valsalva graft and frozen elephant trunk procedure with a covered stent graft were performed if the extent of aortic dissection involved the aortic root and descending thoracic aorta, respectively. After undergoing surgical repair for ATAAD, all patients were transferred to a specialized cardiovascular intensive care unit (ICU) for further treatment and observation. Without unstable haemodynamics, persistent arrhythmia, signs of organ malperfusion, or active bleeding, a ventilator-weaning protocol was initiated at 12–24 h post surgery. Renal replacement therapy was applied according to the Acute Kidney Injury Network criteria if acute renal failure developed after surgery^12^.

### Statistical analyses

Statistical analyses were performed using SPSS for Windows (version 22.0; IBM Corp., Armonk, NY, USA) and SAS for Windows (version 9.4; SAS Institute Inc., Cary, North Carolina, USA). Data were presented as means ± standard deviation for continuous variables and as numbers (n) and percentages (%) for categorical variables. For comparing the intergroup disparities between the survivor and non-survivor groups, we used univariate tests, including the independent *t* test for continuous variables and the chi-square or Fisher’s exact test for categorical variables, respectively. The multivariate logistic regression analysis was used to identify the independent predictors of in-hospital mortality. The variables for multivariate logistic regression analysis were first selected from those with a *P* < 0.05 in univariate tests mentioned above and re-tested by the univariate logistic regression method. Continuous variables were dichotomised based on cut-off values determined by receiver operating characteristics curve analyses before conducting to the logistic regression analyses. Given that the number of events (in-hospital mortality) per covariate was ≤ 10 in some variables, we used penalization through data augmentation to perform multivariate logistic regression to avoid sparse data bias^13,14^. The log-*F*(*m,m*) penalized-likelihood method with the shrinkage parameter (*m*) was implemented by fitting a standard logistic regression to a dataset augmented with pseudo-individuals per covariate. The family of log-F priors will shrink the biased estimate of the regression coefficient in the logistic regression towards the prior odds ratio. In this study, we followed the suggestion by Greenland^15^ to set the prior odds ratio being 1 (uncertain direction) for brain stroke; 2 (probably positive) for repeat surgery, CPB time, aortic clamping time, circulatory arrest time, hypothermia temperature, renal failure; and being 4 (probably strong) for retrograde cerebral perfusion, extracorporeal membrane oxygenation (ECMO) support. In contrast, penalization was not performed in covariates with a number of events > 10, including axillary arterial cannulation, isolated ascending aorta replacement, aortic arch replacement, total arch replacement, ACP, and malperfusion-related complications. The Kaplan–Meier method was used to estimate the 3-year cumulative survival and freedom from aortic reoperation rates for the survivor group. In addition, the independent *t* test, Chi-square and Fisher’s exact test were also used to compare the differences between patients with complicated and uncomplicated ATAAD. For all analyses, statistical significance was set at *P* < 0.05.

## Results

### Patient demographics

As illustrated in Table 1, the clinical demographics, comorbidities, preoperative conditions, and clinical presentation were generally homogenous between the survivor and non-survivor groups, except for the rate of repeat surgery with previous cardiac surgery. In the non-survivor group, the prevalence of repeat surgery was higher than that of the survivor group (2.9% vs. 18.2%; *P* < 0.001). Overall, 20.7% of patients were female, and the mean age was 52.9 ± 12.0 years. Hypertension was a prevalent comorbidity that was observed in approximately 70% of patients in both groups. Chest or back pain was the most common clinical presentation, accounting for > 80% of patients in both groups. A total of 16.7% of the patients were diagnosed with intramural haematoma rather than typical aortic dissection.

**Table 1:** Preoperative characteristics according to patient group.

Parameters	Overall (n = 276)	Survivor (n = 243)	Non-survivor (n = 33)	*P-*value
**Clinical demographics**				
Sex (female, n, %)	57, 20.7	52, 21.4	5, 15.2	0.405
Age (years)	52.9 ± 12.0	52.5 ± 12.1	55.9 ± 11.7	0.129
Body mass index (kg/m^2^)	26.3 ± 4.7	26.5 ± 4.8	25.3 ± 3.7	0.188
Hypertension (n, %)	199, 72.1	176, 72.4	23, 69.7	0.743
Diabetes mellitus (n, %)	10, 3.6	8, 3.3	2, 6.1	0.425
Creatinine (mg/dL)	1.4 ± 1.6	1.4 ± 1.7	1.4 ± 0.8	0.850
eGFR (mL/min/1.73 m^2^)	74.2 ± 27.3	75.1 ± 27.0	67.9 ± 29.0	0.157
ESRD (n, %)	6, 2.2	5, 2.1	1, 3.0	0.719
**Preoperative condition**				
SBP (mmHg)	101.0 ± 10.0	101.4 ± 10.1	97.9 ± 8.1	0.062
Repeat surgery (n, %)	13, 4.7	7, 2.9	6, 18.2	< 0.001
Time from ED to OR (hours)	5.6 ± 4.4	5.6 ± 4.6	5.7 ± 2.0	0.869
**Clinical presentation**				
Chest/back pain (n, %)	243, 88.0	214, 88.1	29, 87.9	0.975
AR > moderate (n, %)	47, 17.0	41, 16.5	6, 21.2	0.496
DeBakey type II (n, %)	13, 4.7	13, 5.3	0	0.174
Intramural haematoma (n, %)	46, 16.7	41, 16.9	5, 15.2	0.803

*AR* aortic regurgitation, *eGFR* estimated glomerular filtration rate, *ED* emergency department, *ESRD* end-stage renal disease, *OR* operating room, *SBP* systolic blood pressure.

### Surgical information

Table 2 provides detailed information regarding surgical variables. Femoral arterial cannulation was a common vascular access used for > 90% of patients in both groups, and the rates of additional axillary arterial cannulation and ACP strategy applied during circulatory arrest were lower in the non-survivor group. A higher rate of aortic arch replacement was found in the non-survivor group than in the survivor group. However, there was no inter-group disparity regarding entry tear exclusion. The time spans of CPB, aortic cross-clamping, and circulatory arrest were generally longer in the non-survivor group. A total of 9.8% of the patients required Kerlix packing due to coagulopathy with uncontrolled bleeding. A higher rate of ECMO implementation due to intraoperative myocardial failure was found in the non-survivor group.

**Table 2:** Surgical information according to patient group.

Parameters	Overall (n = 276)	Survivor (n = 243)	Non-survivor (n = 33)	*P-*value
Femoral arterial cannulation (n, %)	264, 95.7	233, 95.9	31, 93.9	0.607
Axillary arterial cannulation (n, %)	249, 90.2	226, 93.0	23, 69.7	< 0.001
Carotid arterial cannulation (n, %)	5, 1.8	4, 1.6	1, 3.0	0.576
**Aortic repair procedures**				
Entry tear exclusion (n, %)	206, 74.6	183, 75.3	23, 69.7	0.487
Root replacement (n, %)	36, 13.0	31, 12.8	5, 15.2	0.702
Isolated AsAo replacement (n, %)	159, 57.6	146, 60.1	13, 39.4	0.024
Arch replacement (n, %)	88, 31.9	72, 29.6	16, 48.5	0.029
Partial arch (n, %)	47, 17.0	43, 17.7	4, 12.1	0.424
Total arch (n, %)	40, 14.5	28, 11.5	12, 36.4	< 0.001
Frozen elephant trunk (n, %)	26, 9.4	21, 8.6	5, 15.2	0.230
Cardiopulmonary bypass time (min)	259.5 ± 76.6	250.4 ± 64.4	326.9 ± 117.1	0.001
Aortic clamping time (min)	170.1 ± 57.8	165.0 ± 52.0	207.9 ± 80.8	0.005
Circulatory arrest time (min)	53.9 ± 27.7	50.9 ± 21.8	76.0 ± 49.1	0.006
HTK cardioplegic solution (n, %)	181, 65.6	164, 67.5	17, 51.5	0.070
ACP (n, %)	253, 91.7	230, 94.7	23, 69.7	< 0.001
RCP (n, %)	23, 8.3	13, 5.3	10, 30.3	< 0.001
Hypothermia temperature (°C)	20.4 ± 3.1	20.6 ± 3.1	19.3 ± 2.30	0.027
Delayed sternum closure^a^ (n, %)	27, 9.8	25, 10.3	2, 6.1	0.443
ECMO support (n, %)	4, 1.5	1, 0.4	3, 9.1	< 0.001

*ACP* antegrade cerebral perfusion, *AsAo* ascending aorta, *ECMO* extracorporeal membrane oxygenation, *RCP* retrograde cerebral perfusion.

^a^Kerlix packing for uncontrolled coagulopathy and planned secondary exploration.

### Postoperative complications

**Figure 3:**
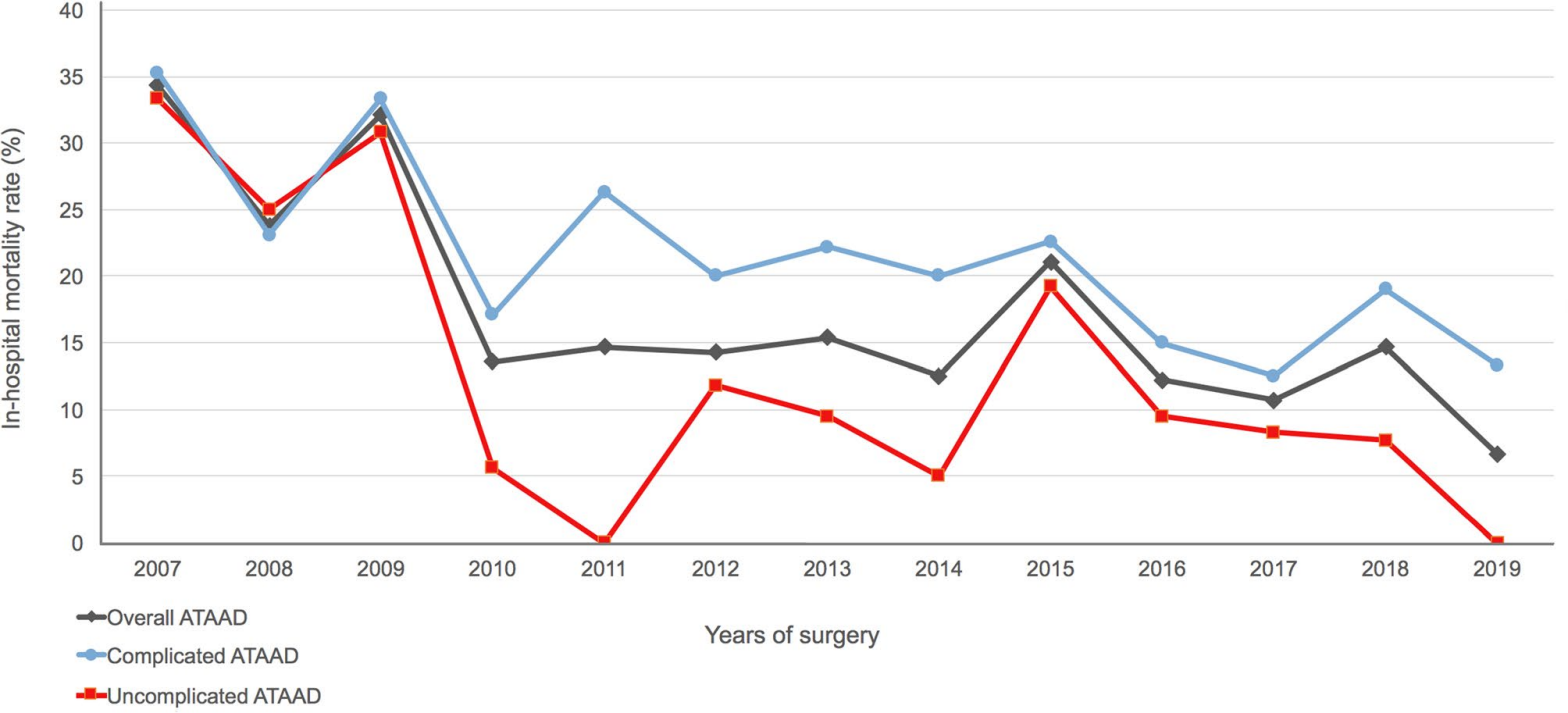
Annual in-hospital mortality rates in overall, complicated, and uncomplicated ATAAD patients from January 2007 to June 2019. ATAAD, acute type A aortic dissection.

The overall in-hospital mortality rate was 11.9%. The annual in-hospital mortality rates in overall, complicated, and uncomplicated ATAAD patients during the study period are illustrated in Fig. 3. In general, patients with uncomplicated ATAAD showed lower annual in-hospital mortality rates compared to that in the overall and complicated populations. By dividing the study group into the first (2007–2010, n = 62) and second periods (2011–2019, n = 214), the in-hospital mortality rates in two periods were 22.6% (14/62) and 8.9% (19/214), respectively. As illustrated in Table 3, myocardial failure was the most common cause of mortality (39.4%), followed by brain stem failure (24.2%), sepsis (21.2%), and bleeding (15.2%). The blood transfusion volumes were generally similar in both groups. In the non-survivor group, malperfusion-related complication rates including postoperative renal failure and brain stroke were significantly higher compared to the survivor group. The overall mean duration of the ICU stay and hospital stay were 7.9 ± 19.1 and 29.5 ± 62.9 days, respectively.

**Table 3:** Postoperative mortality and morbidity according to patient group.

Parameters	Overall (n = 276)	Survivor (n = 243)	Non-survivor (n = 33)	*P-*value
**Etiology of in-hospital mortality**				
Bleeding (n, %)	–	–	5, 15.2	N/A
Myocardial failure (n, %)	–	–	13, 39.4	N/A
Brain stem failure (n, %)	–	–	8, 24.2	N/A
Sepsis (n, %)	–	–	7, 21.2	N/A
Renal failure (n, %)	23, 8.3	13, 5.3	10, 30.3	< 0.001
**Transfusion at 24 h after surgery**				
RBC^a^ (units)	7.7 ± 5.3	7.6 ± 4.8	8.7 ± 8.3	0.432
Plasma^b^ (units)	6.8 ± 4.2	6.6 ± 3.6	8.4 ± 7.2	0.184
Platelet (units)	17.4 ± 10.4	17.3 ± 10.1	18.2 ± 12.3	0.613
Reoperation for bleeding (n, %)	34, 12.3	29, 11.9	5, 15.2	0.598
Atrial fibrillation (n, %)	16, 5.8	13, 5.3	3, 9.1	0.388
Brain stroke (n, %)	43, 15.6	34, 14.0	9, 27.3	0.048
Infarction (n, %)	40, 14.5	32, 13.2	8, 24.2	0.090
Haemorrhage (n, %)	6, 2.2	4, 1.6	2, 6.1	0.103
Delirium (n, %)	49, 17.8	43, 17.7	6, 18.2	0.945
Seizure (n, %)	20, 7.2	16, 6.6	4, 12.1	0.250
Visceral ischemia (n, %)	5, 1.8	5, 2.1	0	0.406
Limb ischemia (n, %)	7, 2.5	7, 2.9	0	0.323
Malperfusion-related complication^c^ (n, %)	63, 22.8	47, 19.3	16, 48.5	< 0.001
Pneumonia (n, %)	28, 10.1	25, 10.3	3, 9.1	0.831
Extubation time (hours)	108.5 ± 365.8	96.3 ± 367.9	199.0 ± 342.1	0.130
Ventilator support > 72 h (n, %)	65, 23.6	57, 23.5	8, 24.2	0.921
Tracheostomy (n, %)	12, 4.4	9, 3.7	3, 9.1	0.155
ICU stay (days)	7.9 ± 19.1	7.7 ± 19.6	8.9 ± 15.0	0.735
ICU readmission (n, %)	21, 7.6	17, 7.0	4, 12.1	0.297
Hospital stay (days)	29.5 ± 62.9	31.2 ± 64.9	16.8 ± 45.0	0.112

*ICU *intensive care unit.

^a^Red blood cell transfusion including amount of whole blood and packed red cell concentrate.

^b^Plasma transfusion including amount of fresh-frozen plasma and cryoprecipitate.

^c^Occurrence of postoperative renal failure, brain infarction, visceral ischemia, and limb ischemia.

### Prognostic factors associated with in-hospital mortality

Table 4 shows the logistic regression analysis results for patients who underwent surgical repair for uncomplicated ATAAD. Axillary arterial cannulation and ACP were highly correlated in the aspect of surgical technique (100% of axillary arterial cannulation underwent ACP; 98.3% of ACP was implemented with axillary arterial cannulation). Furthermore, axillary arterial cannulation and ACP were the exact opposite of retrograde cerebral perfusion. To avoid the problem of collinearity among these three variables, axillary arterial cannulation and ACP were not included in the multivariate logistic regression analysis. The following three significant prognostic factors for in-hospital mortality were identified: repeat surgery (adjusted odds ratio (aOR) 3.01, 95% confidence interval (CI) 1.12–8.09, *P* = 0.029), retrograde cerebral perfusion (aOR 6.07, 95% CI 2.55–14.47, *P* < 0.001), and ECMO support post surgery (aOR 6.31, 95% CI 1.97–20.19, *P* = 0.002).

**Table 4:** Logistic regression results for hospital mortality of patients who underwent surgical repair for uncomplicated acute type A aortic dissection.

Parameters	β-Coefficient	Standard error	Odds ratio, 95% CI	*P-*value	Adjusted for sparse data bias
**Univariate logistic regression**					
Repeat surgery	2.014	0.592	7.49 (2.35–23.92)	< 0.001	Before
	1.459	0.465	4.30 (1.73–10.70)	0.002	After
Cardiopulmonary bypass time > 274 min^a^	1.870	0.429	6.49 (2.80–15.03)	< 0.001	Before
	1.567	0.352	4.79 (2.41–9.55)	< 0.001	After
Aortic clamping time > 179 min^b^	1.489	0.402	4.43 (2.02–9.75)	< 0.001	Before
	1.298	0.343	3.66 (1.87–7.17)	< 0.001	After
Circulatory arrest time > 63 min^c^	1.334	0.381	3.80 (1.80–8.01)	< 0.001	Before
	1.190	0.334	3.29 (1.71–6.32)	< 0.001	After
RCP	2.040	0.474	7.69 (3.04–19.48)	< 0.001	Before
	1.618	0.400	6.27 (2.89–13.63)	< 0.001	After
Hypothermia temperature < 22 °C^d^	1.253	0.623	3.50 (1.03–11.87)	0.044	Before
	1.020	0.446	2.77 (1.16–6.64)	0.022	After
ECMO support	3.186	1.171	24.20 (2.44–240.11)	0.007	Before
	1.940	0.581	6.96 (2.23–21.74)	< 0.001	After
Renal failure	2.040	0.474	7.69 (3.04–19.48)	< 0.001	Before
	1.618	0.400	5.04 (2.30–11.04)	< 0.001	After
Brain stroke	0.835	0.432	2.31 (0.99–5.38)	0.053	Before
	0.602	0.379	1.83 (0.85–3.76)	0.112	After
Axillary arterial cannulation	− 1.754	0.455	0.17 (0.07–0.42)	< 0.001	–
Isolated AsAo replacement	− 0.840	0.380	0.43 (0.21–0.91)	0.027	–
Arch replacement	0.804	0.376	2.24 (1.07–4.67)	0.032	–
Total arch	1.479	0.414	4.39 (1.95–9.88)	< 0.001	–
ACP	− 2.040	0.474	0.13 (0.05–0.33)	< 0.001	–
Malperfusion-related complications	1.367	0.384	3.93 (1.85–8.34)	< 0.001	–
**Multivariate logistic regression**					
Repeat surgery	1.614	0.727	5.02 (1.21–20.86)	0.026	Before
	1.101	0.505	3.01 (1.12–8.09)	0.029	After
Cardiopulmonary bypass time > 274 min^a^	1.443	0.739	4.24 (0.99–18.03)	0.051	Before
	0.795	0.459	2.21 (0.90–5.45)	0.084	After
Aortic clamping time > 179 min^b^	0.066	0.707	1.07 (0.27–4.28)	0.925	Before
	0.266	0.455	1.31 (0.54–3.18)	0.559	After
Circulatory arrest time > 63 min^c^	0.570	0.541	1.77 (0.61–5.11)	0.292	Before
	0.423	0.410	1.53 (0.68–3.41)	0.302	After
RCP	2.249	0.602	9.48 (2.92–30.84)	< 0.001	Before
	1.803	0.443	6.07 (2.55–14.47)	< 0.001	After
Hypothermia temperature < 22 °C^d^	0.272	0.760	1.31 (0.29–5.82)	0.721	Before
	0.545	0.506	1.73 (0.64–4.65)	0.281	After
ECMO support	3.143	1.280	23.18 (1.89–284.87)	0.014	Before
	1.842	0.594	6.31 (1.97–20.19)	0.002	After
Renal failure	0.782	0.766	2.19 (0.49–9.81)	0.308	Before
	0.957	0.723	2.60 (0.63–10.74)	0.186	After
Isolated AsAo replacement	0.231	0.877	1.26 (0.23–7.02)	0.792	–
Arch replacement	0.339	0.983	1.40 (0.21–9.63)	0.730	–
Total arch	1.017	0.716	2.76 (0.68–11.25)	0.156	–
Malperfusion-related complications	0.449	0.607	1.57 (0.48–5.14)	0.460	–

*ACP* antegrade cerebral perfusion, *AsAo* ascending aorta, *AUROC* area under the receiver operating characteristics curve, *ECMO* extracorporeal membrane oxygenation, *RCP* retrograde cerebral perfusion.

^a^AUROC 0.736; sensitivity 75.8%; specificity 67.5%; Youden Index 0.433.

^b^AUROC 0.686; sensitivity 69.7%; specificity 65.8%; Youden Index 0.355.

^c^AUROC 0.644; sensitivity 57.6%; specificity 73.7%; Youden Index 0.313.

^d^AUROC 0.616; sensitivity 90.9%; specificity 23.0%; Youden Index 0.139.

### Cumulative 3-year survival and freedom from reoperation rates

**Figure 4:**
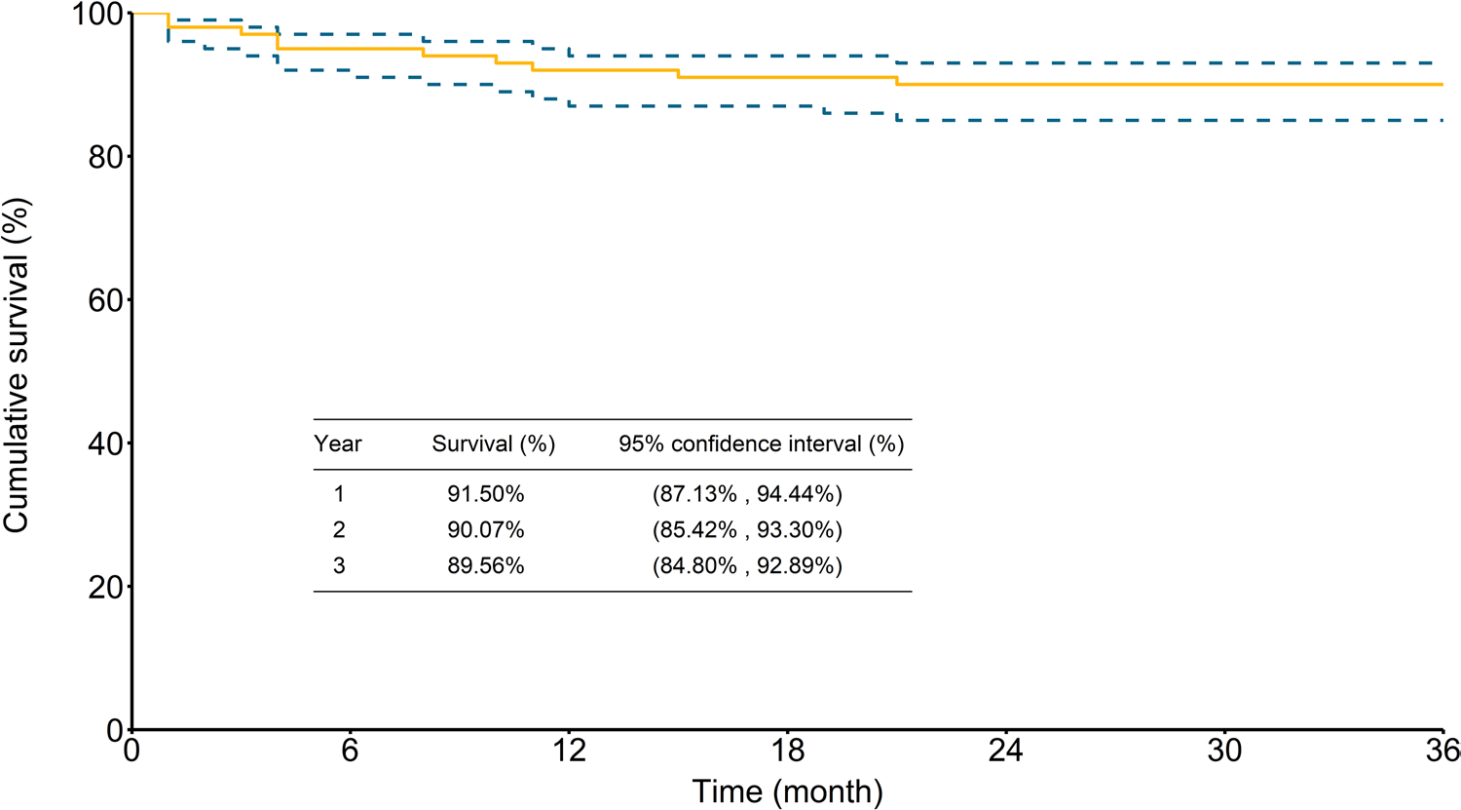
Kaplan–Meier curve of 3-year cumulative survival rate.

**Figure 5:**
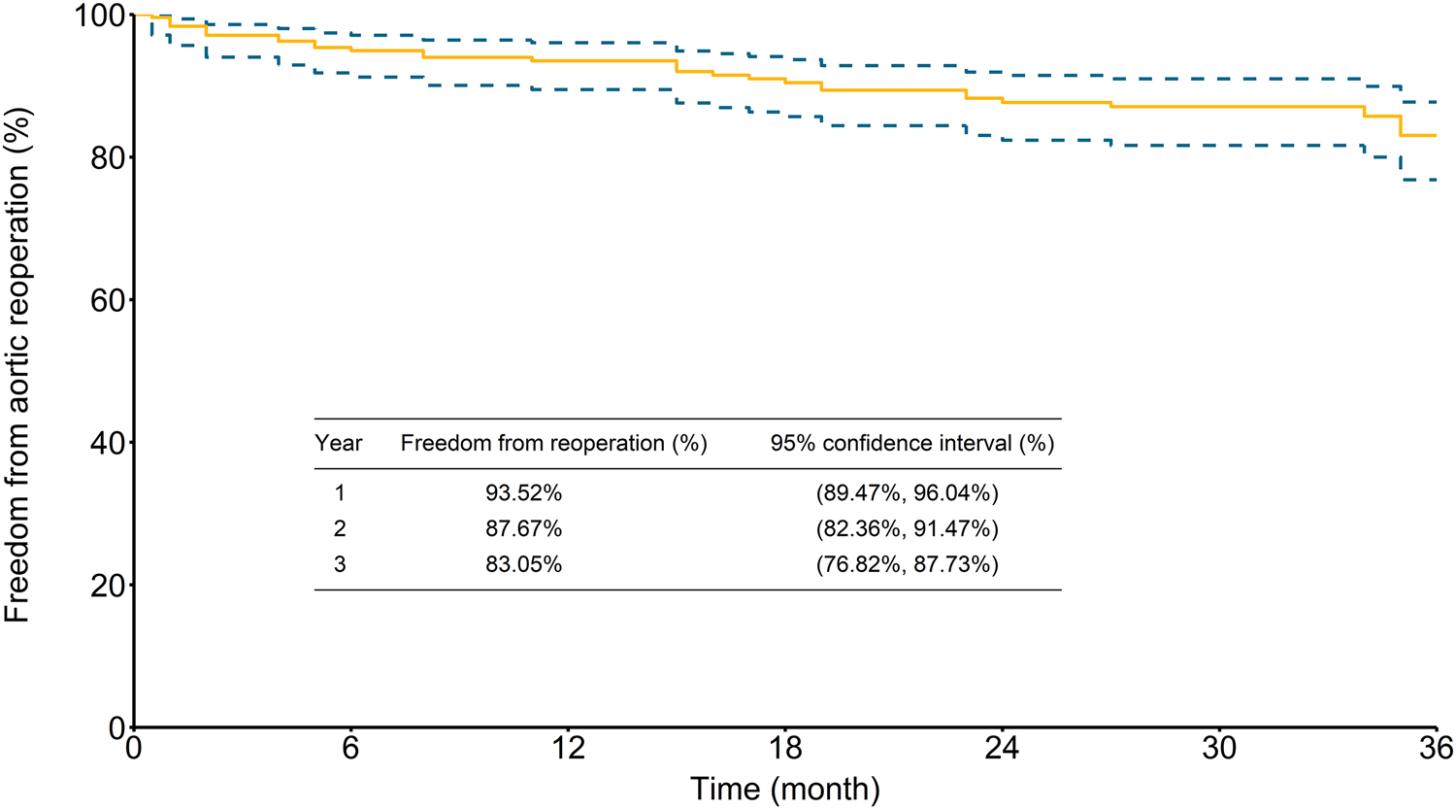
Kaplan–Meier curve of 3-year freedom from aortic reoperation rate.

Follow-up with an average of 4.9 ± 3.5 years (median 4.4, range 0.1–12.7 years) was completed for all patients. As illustrated in Fig. 4, the cumulative survival rates for patients who survived to discharge were 91.5% (95% CI 87.1–94.4%), 90.1% (95% CI 85.4–93.3%), and 89.6% (95% CI 84.8–92.9%) at 1, 2, and 3 years, respectively. As illustrated in Fig. 5, the overall freedom from aortic reoperation rates were 93.5% (95% CI 89.5–96.0%), 87.7% (95% CI 82.4–91.5%), and 83.1% (95% CI 76.8–87.7%) at 1, 2, and 3 years, respectively.

### Comparisons between patients with complicated and uncomplicated ATAAD

As illustrated in Supplementary Table S1, patients with uncomplicated ATAAD were younger with less female compared to the complicated patients. Besides, better preoperative renal function and higher SBP were found in the uncomplicated group than that in the complicated group. In regard to the clinical presentation, the uncomplicated group had a higher rate of typical chest/back pain and a lower incidence of DeBakey type II aortic dissection than the complicated group. Supplementary Table S2 illustrated the surgical information in complicated and uncomplicated ATAAD groups. An higher rate of ACP strategy with a more aggressive aortic arch replacement were found in the uncomplicated group compared with that in the complicated group. As for the postoperative outcomes (Supplementary Table S3), the uncomplicated group showed a lower in-hospital mortality rate (11.9% vs. 20.2%; *P* = 0.007) and fewer blood transfusions than the complicated group. Finally, the subgroup analyses according to the etiologies of complicated ATAAD were illustrated in Supplementary Fig. S4. Patients that were complicated with preoperative shock, CPR and ventilator support showed higher in-hospital mortality rates compared to the uncomplicated ATAAD patients. In addition, patients who had preoperative organ malperfusion and haemopericardium also showed trends of higher in-hospital mortality rates, although the statistical significances were not reached.

## Discussion

In the previous literature, ATAAD with preoperative complications was associated with disastrous outcomes^1–4^. However, the characteristics and surgical results of uncomplicated ATAAD remain to be clarified. Furthermore, the definition and prevalence of uncomplicated ATAAD varied in deferent studies^3,7,16^. In this single-centre study, we excluded most of the preoperative complicated factors that resulted in an inferior outcome, including shock status, organ malperfusion, haemopericardium, ventilator support, and CPR, to obtain a study group of ATAAD patients with the lowest surgical risk. For 276 patients who underwent aortic repair surgery for uncomplicated ATAAD, we found a reasonable in-hospital mortality rate and acceptable mid-term outcomes during the 3-year follow-up. However, it should be possible to optimize the results by improving the surgical strategy and perioperative management.

An expected lower in-hospital mortality rate was observed for the uncomplicated ATAAD patients of this study compared to that reported by previous studies performed at this institute^10,11^, which ranged from 15.5 to 16.0% for the general ATAAD population. Furthermore, as reported by Czemy et al., a large retrospective study in regard to this topic conducted by a national data registry (German Registry for Acute Aortic Dissection Type A database) found that the in-hospital mortality rate in uncomplicated ATAAD patients was 12.6%^3^. A similar outcome was observed in this study. However, the postoperative malperfusion-related complication rates, such as acute renal failure and brain stroke, remained considerable in the present study. We suspected several reasons for this finding. First, malperfusion observed in aortic dissection is correlated to its complex anatomic interactions between the true lumen and the false lumen along the entire dissected aorta. This anatomic interaction can be dynamic during aortic repair surgery. A dissected branch vessel without preoperative malperfusion symptoms may not certainly maintain adequate blood flow to the perfused organ during CPB and circulatory arrest, which render a different blood circulation compared to the normal physiologic pattern. Second, the overall entry tear exclusion rate was only 74.6% in the present study. For patients with residual aortic entry tear, the possibility of true lumen compromise and blood flow limitation may be increased. As reported by Inoue et al., aggressive primary entry tear resection could contribute to the satisfactory short-term and long-term outcomes^17^. We believed that this principle should be applied more aggressively among uncomplicated ATAAD patients because their preoperative condition is relatively stable and they may have better ability to endure a complex surgery. Furthermore, patients with branch vessel dissection but without resection of primary entry tear should be managed with a more aggressive strategy for detecting and treating postoperative malperfusion-related complications early. Finally, approximately 30% of patients underwent aortic arch replacement in this study, and only a total of 3.6% (10/276) of patients underwent bilateral ACP during circulatory arrest. Furthermore, the average circulatory arrest times were > 50 and > 70 min for overall patients and those in the non-survivor group, respectively. The clinical benefits of using bilateral ACP for ATAAD surgery remain controversial^18–20^. However, as reported by Preventza and Angleitner et al., bilateral ACP was associated with superior outcomes for patients requiring prolonged circulatory arrest time > 30 or 50 min^19,20^. Therefore, this modality may also be applied more aggressively to reduce cerebrovascular complications among uncomplicated ATAAD patients included in this study.

In the present study, repeat surgery after previous cardiac surgery was an independent risk factor for in-hospital mortality. Furthermore, the prevalence of repeat surgery in the non-survivor group was six times greater than that in the survivor group. In the previous literatures, repeat cardiac surgery for valvular disease and coronary artery bypass grafting (CABG) could have results comparable to those of first-time surgery performed at experienced centres^21,22^. However, as reported by Rylski and Estrera et al., open surgical repair of ATAAD after previous cardiac surgery was associated with higher in-hospital mortality and brain stroke rates and a more frequent need for postoperative cardiac support compared to first-time surgery^23,24^. Therefore, in the context of ATAAD, it is widely accepted that previous cardiac surgery represents a significant risk factor for poorer outcomes, similar to the results of the present study. We suspect several reasons for this finding. First, for ATAAD patients, all management strategies were performed on an emergent basis, and preoperative evaluation of coronary artery disease, which is essential for patients with previous CABG surgery or scheduled to undergo elective cardiac surgery, was usually inadequate. Therefore, it may increase the risk of insufficient myocardial protection during the redo ATAAD surgery, which usually comprises prolonged CPB and cardiac arrest times. Furthermore, with a less invasive incision compared to resternotomy, left and right thoracotomies could be safe approaches and provide substantial benefits by reducing CPB time and postoperative bleeding for patients undergoing redo CABG and valvular surgeries^25,26^. However, these approaches were usually not considered as a preferable choice for treating ATAAD due to the unsatisfactory exposure of the aortic arch and aortic root. In the present study, all patients underwent aortic repair surgery via sternotomy. Although endovascular stent graft technology is a developing modality that offers an alternative treatment option for selected high-risk patients with ascending aortic disease, including ATAAD^27,28^, it is not feasible for every patient because of the individual differences in the vascular anatomy affected by aortic dissection. Furthermore, the evidence of its long-term durability is lacking. Conventional open repair remains the standard treatment for ATAAD. Finally, to prevent major bleeding from the ruptured aorta when performing resternotomy and dividing the mediastinal adhesions, prior CPB was usually established by peripheral cannulation. However, it could cause significantly prolonged CPB time and increase relative complications.

## Limitations of this study

This study has several limitations. First, as a retrospective and non-randomized control study, bias that could influence the homogeneity of the survivor and non-survivor groups might have existed. Second, because this crossed cohort spans a period of > 12 years, the technology of CPB and myocardial protection, as well as cerebral protection strategies and ICU care protocols for treating ATAAD may have changed over time. Finally, despite the convincing short-term and mid-term results of the present study, an extended follow-up study should be conducted in the future to evaluate long-term outcome in uncomplicated ATAAD population.

## Conclusions

The prevalence of uncomplicated ATAAD was similar to complicated ATAAD, and its short-term and mid-term surgical outcomes were generally acceptable. Even though patients with uncomplicated ATAAD comprise a relatively stable population, accurate surgical strategies for preventing postoperative malperfusion remain crucial to optimize the outcomes.

## Supplementary information

Supplementary Information.

## Data Availability

As containing identifying information, including individual patient’s age, gender, the specific date and details of hospital admission/surgical procedures, the data that we collected cannot be made publicly available for ethical and legal reasons. It is the requirement of the Chang-Gung medical foundation institutional review board to review any request to share data publicly in order to protect patients' privacy. Requests for data can be sent to the corresponding author B9002078@cgmh.org.tw and the Chang-Gung medical foundation institutional review board at irb1@cgmh.org.tw.
