# Supplementary material for: Surgical outcomes analysis in patients with uncomplicated acute type A aortic dissection: a 13-year institutional experience
Source: Sci Rep. 2020 Sep 10;10:14883. doi: 10.1038/s41598-020-71961-4 (PMC7484816; doi:10.1038/s41598-020-71961-4)
Supplement: Supplementary file 1 — Supplementary Information. [file 41598_2020_71961_MOESM1_ESM.doc]

Surgical outcomes analysis in patients with uncomplicated acute type A aortic dissection: A 13-year institutional experience.

Chun-Yu Lin, MD^1^,^2,3*^, Lai-Chu See, PhD^4,5,6^, Chi-Nan Tseng, MD, PhD^1,2^, Meng-Yu Wu, MD^1,2^, Yi Han, BS^4^, Cheng-Hui Lu, MD^1,7^, Feng-Chun Tsai, MD^1,2^

^1^Department of Medicine, College of Medicine, Chang-Gung University, Taoyuan, Taiwan; ^2^Department of Cardiothoracic and Vascular Surgery, Chang-Gung Memorial Hospital, Linkou Medical Centre, Taoyuan, Taiwan; ^3^Department of Cardiothoracic and Vascular Surgery, Chang-Gung Memorial Hospital, Tucheng branch, New Taipei, Taiwan; ^4^Department of Public Health, College of Medicine, Chang-Gung University, Taoyuan, Taiwan; ^5^Biostatistics Core Laboratory, Molecular Medicine Research Centre, Chang-Gung University, Taoyuan, Taiwan; ^6^Division of Rheumatology, Allergy and Immunology, Chang-Gung Memorial Hospital, Linkou Medical Centre, Taoyuan, Taiwan; ^7^Department of Cardiology, Chang-Gung Memorial Hospital, Linkou Medical Centre, Taoyuan, Taiwan.

*Corresponding Author:

Chun-Yu Lin, MD.

Department of Cardiothoracic and Vascular Surgery,

Chang-Gung Memorial Hospital, Linkou Medical Centre,

5 Fu-Shing Street, Kwei-Shan, Taoyuan City, Taiwan 333

Tel: +886-3-3281200; Fax: +886-3-3285818

E-mail: [B9002078@cgmh.org.tw](mailto:B9002078@cgmh.org.tw)

**Supplementary Table S1. Preoperative characteristics in ATAAD patients of the complicated and uncomplicated groups.**

| Parameters | Overall | Complicated | Uncomplicated | *P-*value |
| --- | --- | --- | --- | --- |
|  | (n=603) | (n=327) | (n=276) |  |
| Clinical demographics |  |  |  |  |
| Sex (female, n, %) | 182, 30.2 | 125, 38.2 | 57, 20.7 | <0.001 |
| Age (years) | 56.4 ± 13.9 | 59.3 ± 14.6 | 52.9 ± 12.0 | <0.001 |
| Body mass index (kg/m^2^) | 26.1 ± 4.9 | 25.9 ± 5.1 | 26.3 ± 4.7 | 0.310 |
| Hypertension (n, %) | 427, 70.8 | 228, 69.7 | 199, 72.1 | 0.522 |
| Diabetes mellitus (n, %) | 36, 6.0 | 26, 8.0 | 10, 3.6 | 0.025 |
| Creatinine (mg/dL) | 1.4 ± 1.4 | 1.4 ± 1.3 | 1.4 ± 1.6 | 0.578 |
| eGFR (mL/min/1.73 m^2^) | 69.3 ± 30.9 | 65.1 ± 33.0 | 74.2 ± 27.3 | <0.001 |
| ESRD (n, %) | 11, 1.8 | 5, 1.5 | 6, 2.2 | 0.556 |
| Preoperative condition |  |  |  |  |
| SBP (mmHg) | 94.3 ± 16.5 | 88.7 ± 18.7 | 101.0 ± 10.0 | <0.001 |
| Repeat surgery (n, %) | 25, 4.1 | 12, 3.7 | 13, 4.7 | 0.523 |
| Time from ED to OR (hours) | 5.5 ± 4.4 | 5.4 ± 4.5 | 5.6 ± 4.4 | 0.497 |
| Clinical presentation |  |  |  |  |
| Chest/back pain (n, %) | 451, 74.8 | 208, 63.6 | 243, 88.0 | <0.001 |
| AR > moderate (n, %) | 89, 14.8 | 42, 12.8 | 47, 17.0 | 0.149 |
| DeBakey type II (n, %) | 67, 11.1 | 54, 16.5 | 13, 4.7 | <0.001 |
| Intramural haematoma (n, %) | 108, 17.9 | 62, 19.0 | 46, 16.7 | 0.464 |
| AR, aortic regurgitation; eGFR, estimated glomerular filtration rate; ED, emergency department; ESRD, end-stage renal disease; OR, operating room; SBP, systolic blood pressure. | | | | |

**Supplementary Table S2. Surgical information in ATAAD patients of the complicated and uncomplicated groups.**

| Parameters | Overall | Complicated | Uncomplicated | *P-*value |
| --- | --- | --- | --- | --- |
|  | (n=603) | (n=327) | (n=276) |  |
| Femoral arterial cannulation (n, %) | 579, 96.0 | 315, 96.3 | 264, 95.7 | 0.671 |
| Axillary arterial cannulation (n, %) | 508, 84.2 | 259, 79.2 | 249, 90.2 | <0.001 |
| Carotid arterial cannulation (n, %) | 6, 1.0 | 1, 0.3 | 5, 1.8 | 0.063 |
| Aortic repair procedures |  |  |  |  |
| Entry tear exclusion (n, %) | 442, 73.3 | 236, 72.2 | 206, 74.6 | 0.495 |
| Root replacement (n, %) | 70, 11.6 | 34, 10.4 | 36, 13.0 | 0.312 |
| Isolated AsAo replacement (n, %) | 379, 62.9 | 220, 67.3 | 159, 57.6 | 0.014 |
| Arch replacement (n, %) | 162, 26.9 | 74, 22.6 | 88, 31.9 | 0.011 |
| Partial arch (n, %) | 95, 15.8 | 48, 14.7 | 47, 17.0 | 0.430 |
| Total arch (n, %) | 66, 10.9 | 26, 8.0 | 40, 14.5 | 0.010 |
| Frozen elephant trunk (n, %) | 47, 7.8 | 21, 6.4 | 26, 9.4 | 0.171 |
| Cardiopulmonary bypass time (min) | 259.7 ± 79.8 | 259.9 ± 82.5 | 259.5 ± 76.6 | 0.960 |
| Aortic clamping time (min) | 167.6 ± 56.5 | 165.5 ± 55.5 | 170.1 ± 57.8 | 0.324 |
| Circulatory arrest time (min) | 50.6 ± 25.8 | 47.9 ± 23.7 | 53.9 ± 27.7 | 0.005 |
| HTK cardioplegic solution (n, %) | 381, 63.2 | 200, 61.2 | 181, 65.6 | 0.262 |
| ACP (n, %) | 517, 85.7 | 264, 80.7 | 253, 91.7 | <0.001 |
| RCP (n, %) | 86, 14.3 | 63, 19.3 | 23, 8.3 | <0.001 |
| Hypothermia temperature (°C) | 20.3 ± 2.7 | 20.1 ± 2.3 | 20.4 ± 3.1 | 0.158 |
| Delayed sternum closure^a^ (n, %) | 80, 13.3 | 53, 16.2 | 27, 9.8 | 0.020 |
| ECMO support (n, %) | 17, 2.8 | 13, 4.0 | 4, 1.5 | 0.062 |
| ^a^Kerlix packing for uncontrolled coagulopathy and planned secondary exploration.  ACP, antegrade cerebral perfusion; AsAo, ascending aorta; ECMO, extracorporeal membrane oxygenation; RCP, retrograde cerebral perfusion. | | | | |

**Supplementary Table S3. Postoperative mortality and morbidity in ATAAD patients of the complicated and uncomplicated groups.**

| Parameters | Overall | Complicated | Uncomplicated | *P-*value |
| --- | --- | --- | --- | --- |
|  | (n=603) | (n=327) | (n=276) |  |
| In-hospital mortality (n, %) | 99, 16.4 | 66, 20.2 | 33, 11.9 | 0.007 |
| Bleeding (n, %) | 19, 3.2 | 14, 4.3 | 5, 1.8 | 0.084 |
| Myocardial failure (n, %) | 40, 6.6 | 27, 8.3 | 13, 4.7 | 0.081 |
| Brain stem failure (n, %) | 21, 3.5 | 13, 4.0 | 8, 2.9 | 0.472 |
| Sepsis (n, %) | 20, 3.3 | 13, 4.0 | 7, 2.5 | 0.325 |
| Renal failure (n, %) | 54, 9.0 | 31, 9.5 | 23, 8.3 | 0.623 |
| Transfusion at 24 hours after surgery |  |  |  |  |
| RBC^a^ (units) | 8.6 ± 7.5 | 9.3 ± 8.8 | 7.7 ± 5.3 | 0.007 |
| Plasma^b^ (units) | 7.6 ± 6.4 | 8.2 ± 7.7 | 6.8 ± 4.2 | 0.006 |
| Platelet (units) | 18.2 ± 12.5 | 18.9 ± 14.0 | 17.4 ± 10.4 | 0.118 |
| Reoperation for bleeding (n, %) | 87, 14.4 | 53, 16.2 | 34, 12.3 | 0.176 |
| Atrial fibrillation (n, %) | 36, 6.0 | 20, 6.1 | 16, 5.8 | 0.869 |
| Brain stroke (n, %) | 89, 14.8 | 46, 14.1 | 43, 15.6 | 0.602 |
| Infarction (n, %) | 81, 13.4 | 41, 12.5 | 40, 14.5 | 0.483 |
| Haemorrhage (n, %) | 13, 2.2 | 7, 2.1 | 6, 2.2 | 0.978 |
| Delirium (n, %) | 111, 18.4 | 62, 19.0 | 49, 17.8 | 0.703 |
| Seizure (n, %) | 38, 6.3 | 18, 5.5 | 20, 7.2 | 0.381 |
| Visceral ischemia (n, %) | 15, 2.5 | 10, 3.1 | 5, 1.8 | 0.328 |
| Limb ischemia (n, %) | 19, 3.2 | 12, 3.7 | 7, 2.5 | 0.427 |
| Malperfusion-related complication^c^ (n, %) | 142, 23.5 | 79, 24.2 | 63, 22.8 | 0.701 |
| Pneumonia (n, %) | 68, 11.3 | 40, 12.2 | 28, 10.1 | 0.419 |
| Extubation time (hours) | 98.4 ± 273.2 | 89.9 ± 157.5 | 108.5 ± 365.8 | 0.432 |
| Ventilator support >72 hours (n, %) | 174, 28.9 | 109, 33.3 | 65, 23.6 | 0.008 |
| Tracheostomy (n, %) | 28, 4.6 | 16, 4.9 | 12, 4.4 | 0.751 |
| ICU stay (days) | 7.4 ± 14.6 | 6.9 ± 9.1 | 7.9 ± 19.1 | 0.422 |
| ICU readmission (n, %) | 39, 6.5 | 18, 5.5 | 21, 7.6 | 0.295 |
| Hospital stay (days) | 26.8 ± 46.4 | 24.6 ± 25.0 | 29.5 ± 62.9 | 0.200 |
| ^a^Red blood cell transfusion including amount of whole blood and packed red cell concentrate.  ^b^Plasma transfusion including amount of fresh-frozen plasma and cryoprecipitate.  ^c^Occurrence of postoperative renal failure, brain infarction, visceral ischemia, and limb ischemia.  ICU, intensive care unit. | | | | |

**Supplementary Figure S4. Subgroup analyses of outcomes according to etiologies of complicated ATAAD.**

**
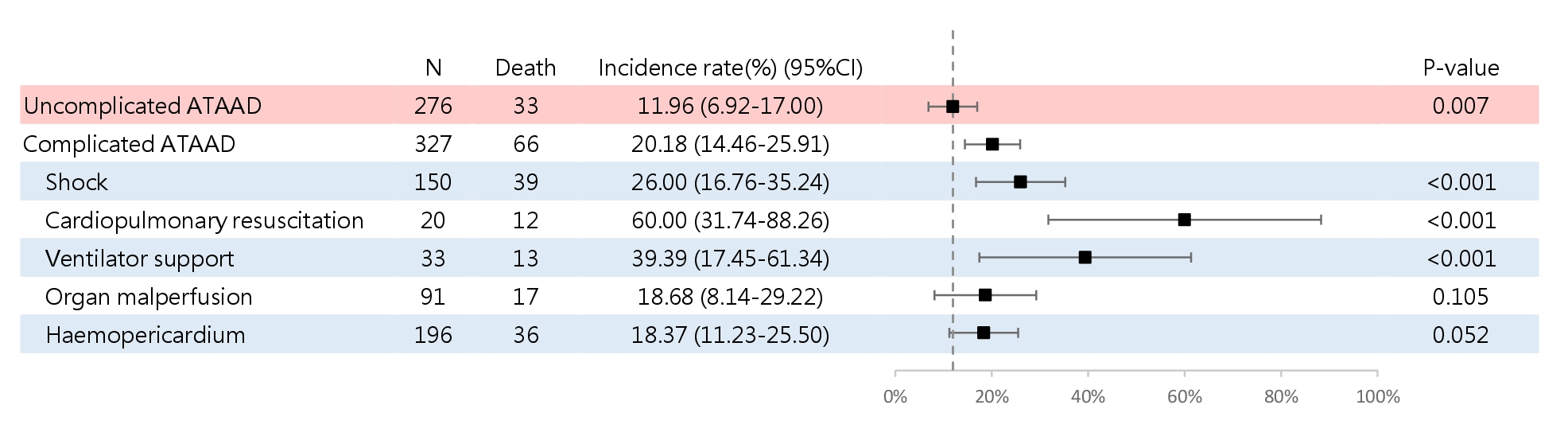
**
